# Response: arguments to abolish the legal age limits of access to information about the gamete donor by donor offspring

**DOI:** 10.1136/jme-2024-110230

**Published:** 2024-07-26

**Authors:** Inge van Nistelrooij, Nicolette Woestenburg

**Affiliations:** 1 Department of Care Ethics, University of Humanistic Studies, Utrecht, The Netherlands; 2 Pro Facto, Groningen, The Netherlands

**Keywords:** Ethics, Ethics- Medical, Human Rights, Policy, Reproductive Medicine

## Abstract

The *Journal of Medical Ethics* previously published on the debate in the UK and the Netherlands concerning the legal age limits imposed on donor-conceived people for access to information about the identity of gamete and embryo donors. In that publication, three arguments were foregrounded against lowering these age limits as a general rule for all donor-conceived people. In this contribution, we engage with these arguments and argue why we think they are insufficient to maintain the age limits. In contrast, we argue for a more suited, contextual and relational ethical framework based on care ethics, which emphasises relational autonomy and its dynamic, contextual development. This framework, we argue, provides a comprehensive approach for the analysis we made of the question of age limits and was applied in research performed in the Netherlands, commissioned by the Dutch Minister of Health. The framework enabled us to weigh the multidisciplinary—legal, psychological, phenomenological and ethical—findings of our research.

## Introduction

 The *Journal of Medical Ethics* recently published^1^ on the debate in the UK and the Netherlands concerning the legal age limits imposed on donor-conceived people for access to the identity of gamete and embryo donors. In it, reference is made to the advice of our research performed in the Netherlands,^2^,^5^ commissioned by the Dutch Minister of Health, to abolish these age limits in order to enable families to develop a suited, relational story for children in their development towards autonomy. Currently, the legal age for donor conceived people to request information about the donor identity is 16, a child aged 12 could request information on physical and social characteristics of the donor, like eye colour and level of education. Our advice to abolish the legal age limits was the result of a multidisciplinary research performed by researchers from three disciplines and organisations—ethicists (University of Humanistic Studies), legal scientists (Pro Facto) and psychosocial scientists (Fiom)—and two experiential experts: a donor-conceived person and a donor who is also a donor-conceived person. The elaborate advice was based on all findings of the multidisciplinary research, which we have elaborated elsewhere.^2^,^5^ It is important to note that our research question is broader than the ethical underpinning of age limits and also looks into carefully handling of providing donor information. By formulating conditions for providing donor information the consortium did not restrict itself to moral grounds for age limits but broadened its scope to the ethical question of building a good society in which donor-conceived people, their families and relatives can flourish.

In what follows, we will respond to the arguments offered by Pennings^1^ against lowering age limits, referring to the care ethical framework we used in our research, which we deem more adequate for addressing the question of age limits than the medical ethical framework from which the age limits were adopted in the Netherlands. This care ethical framework is a more suited, contextual and relational ethical framework. Care ethics allows for an ethical weighing of moral goods as they emerge in lived experiences, collective practices of care, and institutional, legal and political regulations of those practices.^6^ In our research, we argue for a family setting where the child’s life story can be narrated in which donor conception and information can be interwoven, rather than a general, inflexible right to information from a fixed age limit. For a more comprehensive legal and ethical underpinning of our advice, we refer to our article in Medical Humanities^5^ and the findings of our multidisciplinary research.^2^,^4^

## Responses to the arguments against lowering age limits

Pennings^1^ offers three arguments against lowering the age limits as a general rule for all donor-conceived people, in a contribution focused on the question ‘whether one should give a child the right to obtain the identity of the donor at an earlier age than is presently stipulated’.^1^ We present Pennings’ arguments and our responses to them.

### Argument 1: discussion of the argument of lacking evidence

Pennings argues ‘that there is no evidence that a change in age will increase the total well-being of the donor offspring as a group’. The ethical demand for evidence for an increased well-being is strange in itself, for as a direct consequence of the age limits, the group that could provide information on their increased well-being as a result from a change of age limits for access to donor information is non-existent. The age limits have precluded younger children and their parents to access donor information, therefore, no such evidence can be retrieved. Indeed, no argument can be made that lowering (or abolishing) the age limits will definitively lead to increased or decreased well-being based on evidence. Instead, the only information available is on experiences with the present age limits and their effect on the well-being of those involved.

Pennings^1^ contends that ‘(t)he HFEA considers lowering the age due to direct-to-consumer genetic testing that enables donors, recipients and donor-conceived people to identify genetic relatives outside the legal framework’. This was not the case in the Netherlands, where research on the current age limits started from the determination that the law lacked an adequate underpinning for them: there were no arguments provided in the first place for the prohibition of access to donor information.^7^ We have embedded our ethical, psychological and empirical findings in the legal development of increased acknowledgement that access to donor information is underpinned by the international right of children to know their own parents (article 7 of the Convention of the Rights of the Child) and the right to preserve their identity, including name, nationality and family relations (article 8 of the Convention).

DNA-testing, however, is also important in the Dutch discussion because laws should be enforceable, otherwise the legal ground of a law is lacking. If people can find donor information through DNA tests without any limitation, this affects the assumption of the legislator that laws offer certainty and equality, which is an important factor in constituting a law. The social developments and increased digitalisation, since this law was constituted, should be taken into account when reviewing the age limits.

Pennings’ argument continues with the plea that primary responsibility should be taken ‘to discourage people from having these DNA tests or at least to warn the consumers for unexpected findings and misconceptions about the certainty of these tests’. We do not believe that a campaign discouraging DNA tests can be very successful, first as it denies the reality of digital answers to ‘consumer wishes’, but second since we believe that there is more at stake. We estimate that online DNA tests meet a real need. Donor-conceived people have, for example, expressed their worries about having sexual relationships with siblings.^2^ We, therefore, uphold the human right of access to ancestry information, as it adequately addresses their concrete concerns.

Pennings argues that some children will benefit from receiving the information earlier, but ‘other children will be harmed by the knowledge of and/or contact with the donor.’ Pennings does not argue how accessibility of information (for which some will be interested, others ambivalent or not interested) leads to accessible information itself being harmful. Making the information accessible is not the same as forcing information on people and does not preclude donor-conceived children who are ambivalent or not interested to simply ignore the option. This should not impede those who have an explicit need for it, to access ancestry information. Nor is accessibility of information the same as having contact, nor is contact an automatic result, as all parties still have to give consent; a misconception that Pennings repeats later on. Contrary to Pennings’ accusation of failure on our behalf (‘The report ordered by the Dutch authorities defends abolishing all age limits but fails to explain how the practice should be organised then‘), we have provided ample advice on how to provide for a practice that carefully handles this information and supports parents. Rather than a general prohibition for all, we propose that closely engaged others (like parents) are allowed to provide information that is attuned to the actual expressed need, and that they are supported by state-of-the-art information and suited counselling.

### Argument 2: discussion of the argument of isolation of the child caused by a logic of rights

Pennings’ second set of arguments revolves around the logic of rights which causes the child to be treated as isolated from the relational setting in which it grows up. We completely agree with his argument that ‘by taking a ‘rights’ perspective one isolates the right holder (ie, the child), from his/her social environment’. What Pennings fails to see, however, is that a plea for keeping the age limits in place has the exact same effect. For general age limits also separate the donor-conceived child from their parents, disabling them to play any active role in catering to the needs of the child to know more about the donor until that age.

Second, Pennings argues that ‘this would constitute a serious violation of the right of the parents to decide what measures should be taken for the well-being of their children. An expert may decide that their child is competent while they may disagree or object on the basis of other arguments such as the reaction of a younger sibling.’ This argument also fits well with the arguments in our advice: we argue for the right to have information and to have the information embedded in the relational context of the family. Making the information available to the parents at any time they wish, enables them to handle it when needed, attuning to each child’s interest. This may lead to a young child wanting to know if the donor also likes football, or to give no information when the child does not want it. We also argue that support and counselling by professionals is provided throughout the child’s upbringing, as part of the Dutch youth healthcare chain—consisting of the child health clinic (‘consultatiebureau’), school nurses and doctors—that already supports parents. A decontextualised and individualising right is contrary to what we have argued for.

Pennings’ argument that the parents may prefer ‘an unimpeded family life up to the child’s maturity’ reintroduces the argument previously refuted: that giving information depends on the child reaching a certain, and generally valid, age. We take a different view and argue that the larger vulnerability of the donor-conceived person compared with the vulnerability of adults leads to a larger responsibility of the parents regarding their children. We argue against a primary responsibility of the government to impact all families with a general rule.

And finally, the argument that ‘early contact may represent a risk for clear psychological boundaries’ and may be a risk for ‘a stable relationship with the parents’ may also be valid when reversed: openness, trust and reliability may be the best grounds for stable relationships, as opposed to the harm caused by secrecy and finding out that one has been misinformed. Instead, having information on a donor that is interwoven with the narratives about one’s own life may help establish clear psychological boundaries embedded in relations, in the sense of knowing where talents or looks come from.^2 4^ Again Pennings refers to having contact, but it remains essential to distinguish between information and contact: accessing information is no direct cause for concern regarding contact or involvement.

### Argument 3: discussion of the argument of bionormative ideology

Pennings^1^ accuses those advocating the lowering of age limits of fitting ‘well into the bionormative ideology of the family’, an ideology that is ‘on the rise’ as is shown by the increased popularity of ‘ancestry tests’ and ‘by the increase in countries abolishing donor anonymity’.

We agree that for many donor-conceived children, having information on the identity of the donor is necessary for identity construction. We, therefore, plea for the accessibility of this information and indeed, we consider identity building a lifelong process, which takes place from an early age, and which requires that the information can be interwoven in this process. However, we believe that the information should be available rather than forced on the child and have stressed that personal attunement to the child’s needs is paramount.^2^

Does this make us adherents of a bionormative ideology? We believe not. We have not argued for an essentialist view on ancestry. We have instead argued against general inaccessibility of this information until a certain age. Making information accessible is neither stating that it is essential to know this information, nor that additional contact is essential. Sometimes, it is helpful to simply know some features (hair colour) or characteristics (education level) so that one knows why one differs from family members. We also argue for various forms of that information. That is our plea: to have the option to receive information, nothing more, nothing less.^2^

We also reject the idea that we adhere to an ideology that ‘maintains the superiority of families in which parents and children are genetically related’.^1^ It is important to make our normative view clear here: when scrutinising arguments that underpin the age limits, we have started from the ethical standpoint of listening to the needs of those who are involved, not to judge on any ideological grounds. We thereby adopt a care ethical moral epistemology.

## Conclusion and advice

We have argued that many of the arguments against lowering (or abolishing) legal age limits for accessing donor information presented in the *Journal of Medical Ethics* have not been adequate. These arguments support the current legal age limits but do not address the weak reasoning on which they were based. We suggested to adopt a care-ethical approach to looking at the question of accessibility of donor information. This contextual, relational ethical framework based on care ethics, which emphasises relational autonomy and its dynamic, contextual development, is more suited for the question of age limits. In our research, we argue for a primary responsibility of the parents to interweave donor conception and information in the child’s life story to cater to their needs, rather than a general, inflexible right to information from a fixed age limit.
